# The relationship between parental-child attachment consistency and shyness in adolescents with depressive disorders

**DOI:** 10.3389/fpsyg.2026.1781701

**Published:** 2026-06-12

**Authors:** Mengyuan Zhang, Furong Gou, Xinrong Ma, Ling Dang, Yuelan Zhang, Liqi Gu, Xin Tian

**Affiliations:** 1 School of Nursing, Ningxia Medical University, Yinchuan, Ningxia, China; 2 Department of Psychiatry and Clinical Psychology, Ningxia Ning-An Hospital, Ningxia Mental Health Center, Yinchuan, Ningxia, China; 3 Department of Emergency Medicine, Shanxi Academy of Medical Sciences, Shanxi Bethune Hospital, Tongji Shanxi Hospital, Third Hospital of Shanxi Medical University, Taiyuan, Shanxi, China

**Keywords:** adolescents with depressive disorders, maternal attachment, paternal attachment, shyness, social-emotional adaptation

## Abstract

**Background:**

Shyness is a common socio-emotional problem among adolescents with depressive disorders, and parent–child attachment may be associated with adolescents’ social functioning. However, the independent and combined effects of maternal and paternal attachment on shyness remain unclear.

**Methods:**

This cross-sectional study included 252 adolescents diagnosed with depressive disorders in Ningxia, China. Questionnaires were used to assess maternal and paternal attachment security and shyness. Polynomial regression combined with response surface analysis was applied to examine the associations between maternal and paternal attachment consistency and shyness, controlling for gender.

**Results:**

Both maternal and paternal attachment were significantly negatively correlated with shyness, indicating that higher attachment security was associated with lower shyness levels. Response surface analysis revealed that adolescents exhibited the lowest levels of shyness when both maternal and paternal attachment were high and consistent. Differences between parental attachment were not significantly associated with shyness.

**Conclusion:**

The findings suggest that secure maternal and paternal attachments are associated with lower levels of shyness in adolescents with depressive disorders. High and consistent parental attachment was associated with a more stable emotional context, which was associated with adolescents’ social confidence and emotion regulation. Interventions should focus on improving both the quality and consistency of parental attachment in relation to adolescent shyness and socio-emotional adaptation.

## 1 Introduction

Depressive disorder is one of the most prevalent and debilitating mental health conditions affecting adolescents. It is primarily characterised by persistent low mood, reduced interest, and a loss of pleasure (Lu et al., 2024). Due to its early onset and high recurrence rate, it has become a major global public health concern. According to the World Health Organization, the global prevalence of depression among adolescents aged 10–19 is 4.9%, making it the leading cause of illness and disability in this age group (World Health Organization, 2025). In China, the China National Mental Health Development Report (2021–2022) indicates that the detection rate of depressive symptoms among adolescents is as high as 18.6%. As research in this area has expanded, increasing attention has been paid to the high prevalence of shyness among adolescents with depressive disorders and its potential clinical significance. Shyness is defined as a tendency to experience tension, fear, and discomfort in social situations, particularly when individuals feel they are being evaluated. This often leads to social inhibition and withdrawal (Bowker et al., 2019). Studies have shown that adolescents with depressive disorders tend to report higher levels of shyness, and that greater shyness is associated with poorer social functioning (Cheng et al., 2025). These findings suggest that shyness may be an important manifestation of social adaptation difficulties in this population (Wu et al., 2024). Among the various factors related to shyness, parent–child attachment has been widely identified as a key correlate (Krieg and Dickie, 2013; Zimmermann et al., 2015). However, most previous studies have examined parent–child attachment as a single, global construct. In contrast, maternal and paternal attachment may have distinct associations with adolescents’ shyness. Moreover, little is known about how the consistency between maternal and paternal attachment is associated with shyness. To address these gaps, the present study focuses on adolescents with depressive disorders. It separately examines the associations between maternal and paternal attachment and shyness and further investigates the role of their consistency. This study aims to provide new insights into the psychological correlates of shyness in adolescents with depressive disorders and to offer empirical evidence relevant to family-based intervention strategies.

### 1.1 Parent–child attachment and shyness in adolescents with depressive disorders

Parent–child attachment refers to stable emotional bonds formed through ongoing interactions with parents. These bonds provide a crucial psychological foundation for the development of emotional security and social support in children and adolescents (Ali et al., 2021). Core features of attachment relationships include trust, emotional availability, and a sense of security during times of stress. According to attachment theory, sensitive responsiveness and emotional support from parents in early childhood are associated with later social behavior patterns and emotional responses (Ainsworth, 1997). When children lack stable and secure attachment experiences, they are more likely to develop tension, defensiveness, and excessive self-focus in social situations (Tang and Schmidt, 2017). These characteristics are associated with the psychological features relevant to shyness. Therefore, parent–child attachment is not only fundamental to social development but also is associated with adolescent shyness. Although relatively few studies have examined shyness as a primary outcome, a substantial body of research has linked parent–child attachment to related constructs such as social anxiety and social withdrawal in adolescence (Muzi et al., 2022; Xiao et al., 2025). It is important to distinguish these constructs. Shyness refers to a relatively stable dispositional tendency toward inhibition in social situations (Bowker et al., 2019). Social anxiety involves a more intense and persistent fear of negative evaluation (Wu et al., 2024). Social withdrawal refers to observable reductions in social interaction. Despite these conceptual distinctions (Masi et al., 2023), the three constructs overlap in their emotional and behavioral manifestations (Watling, 2015). Empirical studies further support the association between parent–child attachment and these related outcomes. For example, Carcedo et al. (2023) found that parent–child attachment was associated with friendship quality and loneliness during the transition to college through social anxiety. This finding suggests that secure attachment may be associated with sensitivity to social evaluation and interpersonal avoidance (Carcedo et al., 2023). Similarly, El-Sayed and Mwangi (2025) reported that higher-quality parent–child attachment was associated with lower levels of social anxiety in a Kenyan adolescent sample, and this association was strengthened by perceived peer rejection. In addition, meta-analytic evidence indicates that both maternal and paternal attachment and parenting styles are significantly related to adolescents’ social anxiety (Howard et al., 2025). Among these factors, maternal warmth and responsiveness appear to show a particularly strong association with avoidance and shyness tendencies (Howard et al., 2025). Taken together, these findings suggest that secure parent–child attachment is associated with adolescents’ sense of security and self-confidence in social interactions, which may be associated with negative emotional responses such as social anxiety and shyness. Although most existing studies focus on general adolescent populations, the influence of attachment may be even more pronounced among adolescents with depressive disorders, given their difficulties in emotional regulation.

### 1.2 From the perspective of maternal and paternal attachment: a response surface analysis approach

It is worth noting that most existing studies examining the relationship between parent–child attachment and adolescent shyness tend to treat attachment as a single, unified construct. This approach overlooks the functional and psychological differences between maternal and paternal attachment (Pinquart, 2023a). According to family systems theory, the family is an interdependent and dynamically interacting system. Emotional bonds between parents and children do not operate in isolation; rather, they jointly shape adolescents’ psychological and behavioral development through ongoing interactions (Rothbaum et al., 2002). Therefore, considering both maternal and paternal attachment, as well as their consistency, is essential for a more comprehensive understanding of parent–child relationship dynamics within the family system. Within this framework, polynomial regression and response surface analysis provide a useful methodological approach that addresses the limitations of traditional linear models. This method allows researchers to examine the independent associations of maternal and paternal attachment while also capturing their combined association with shyness through a three-dimensional response surface. In doing so, it illustrates both interaction and consistency effects (Shanock et al., 2010). More importantly, this approach enables the examination of how adolescents’ levels of shyness vary under conditions of high consistency or marked discrepancy between parental attachment levels. As such, it extends beyond traditional analytical perspectives and offers new methodological insights into the complex association between parent–child attachment and shyness. Furthermore, a growing body of research suggests that maternal and paternal attachment may play distinct roles in adolescents’ socio-emotional development (Leben Novak et al., 2023; Iwanski et al., 2021; Dagan et al., 2021). Maternal attachment is typically associated with emotional support and a sense of security, and is often discussed as a key resource for emotion regulation and psychological comfort in stressful situations (Pinquart, 2023b). In contrast, paternal attachment is more strongly associated with the development of autonomy, exploration, and self-efficacy, and is considered to contribute to adolescents’ social adaptation (Olsavsky et al., 2020). At the same time, adolescents are typically reported to perceive their mothers as their primary emotional attachment figures, while regarding their fathers as important sources of behavioral guidance and autonomy support (Allen et al., 2024; Dennis et al., 2025).

The relationship between parent–child attachment consistency and adolescent shyness remains unclear. The attachment congruence hypothesis provides a useful theoretical framework for understanding this association. Proposed by Cowan within the framework of family systems theory, this hypothesis emphasizes that the degree of consistency between maternal and paternal attachment security plays an important role in children’s psychosocial development (Cowan and Cowan, 2002). Its core assumption is that children are influenced not only by their individual attachment relationships with each parent, but also by the overall coherence or inconsistency between parental attachment styles. Together, these factors are considered to shape the emotional climate of the family (Cowan and Cowan, 2002). From a family systems perspective, mothers and fathers function as interdependent subsystems, and the consistency of their attachment security is associated with a more stable and predictable caregiving environment. When both parents demonstrate high and consistent attachment security, children are more likely to experience coherent emotional support, stable caregiving responses, and reduced relational ambiguity. This consistency is considered to facilitate the development of a more stable internal working model characterized by positive expectations of both the self and others (Dykas and Cassidy, 2011). Such a coherent internal working model is further associated with emotional regulation capacity and interpersonal confidence, and may be related to lower self-consciousness and behavioral inhibition in social contexts (Kochanska and Kim, 2013). In contrast, inconsistency between maternal and paternal attachment security may be associated with mixed interpersonal signals and emotional uncertainty within the family system. This lack of coherence may undermine children’s sense of relational security and increase vigilance toward social evaluation, thereby strengthening tendencies toward interpersonal inhibition. These processes are considered to be related to the development of shyness. Although direct evidence linking parental attachment consistency to shyness in adolescents with depressive disorders remains limited, related findings provide indirect support. van Eijck et al. (2012) found that adolescents’ perceived parent–child attachment quality was negatively associated with anxiety and social withdrawal. Similarly, Parade et al. (2010) reported that secure parental attachment was associated with lower levels of social anxiety and better peer relationship functioning among university freshmen. Zhao et al. (2025), in a longitudinal study of junior high school students, found that poor parent–child relationships were associated with higher levels of shyness and increased vulnerability to adverse social outcomes, such as cyber victimization. Taken together, these findings suggest that parental attachment consistency may be associated with adolescent shyness by shaping the coherence of the family emotional environment and the stability of internal working models. This association may be particularly pronounced in adolescents with depressive disorders, who tend to be more sensitive to interpersonal stress and emotional inconsistency due to difficulties in emotion regulation.

### 1.3 Current study

Although an increasing number of studies have identified an association between parent–child attachment and adolescent shyness, most research has focused on the general adolescent population. Empirical evidence specifically targeting adolescents with depressive disorders remains limited. This group often experiences difficulties in emotion regulation, self-concept, and social interaction, suggesting that their shyness may be associated with family attachment security. In addition, existing studies typically treat parent–child attachment as a single, integrated construct and rarely distinguish between the independent associations of maternal and paternal attachment. They also tend to overlook the potential interactive association of consistency or inconsistency between the two with adolescent shyness. Traditional analytical approaches further struggle to capture the complex nonlinear associations related to parental attachment consistency. To address these gaps, the present study adopts a dual-perspective approach by examining both maternal and paternal attachment among adolescents with depressive disorders. Using polynomial regression and response surface analysis, it investigates the patterns of association between parental attachment and shyness. Based on attachment theory and existing empirical evidence, the following hypotheses are proposed:

*H1*: Both paternal attachment security and maternal attachment security are significantly negatively associated with shyness levels in adolescents with depressive disorders, meaning that higher parental attachment security is associated with lower levels of shyness in adolescents.

*H2*: When both paternal and maternal attachment security are at higher levels, adolescents with depressive disorders are expected to exhibit lower levels of shyness.

## 2 Method

### 2.1 Participants

This study employed a cross-sectional design and convenience sampling method to recruit adolescents with depressive disorders from both the outpatient and inpatient departments of Ning’an Hospital, Ningxia Hui Autonomous Region, China, between June 2024 and March 2025. Participants met the diagnostic criteria for depressive disorders as defined in the Diagnostic and Statistical Manual of Mental Disorders, Fifth Edition (DSM-5). DSM-5 diagnoses were established using the Structured Clinical Interview for DSM-5 Disorders (SCID-5), conducted by two independent psychiatrists (each holding at least the qualification of attending physician). Each diagnosis was confirmed through consensus between the two psychiatrists based on the SCID-5 interview and clinical judgment. Eligible participants were aged 12 to 18 years, provided written informed consent together with their legal guardians, and possessed sufficient reading and comprehension ability to complete the questionnaires independently or under supervision. Individuals with other severe psychiatric conditions (e.g., schizophrenia, bipolar disorder, organic mental disorders, developmental disorders, or substance use disorders) or with serious physical or neurological diseases that might affect psychological assessment were excluded. Prior to data collection, all participants and their guardians signed informed consent forms. After excluding invalid or incomplete questionnaires, a total of 252 valid responses were obtained, including 55 males (21.8%) and 197 females (78.2%), with a mean age of 15.44 ± 1.69 years. The study protocol was reviewed and approved by the Ethics Committee of Ning’an Hospital (Approval No. 2023-WS-022).

### 2.2 Measurement

#### 2.2.1 Parent–child attachment

This study employed the Inventory of Parent Attachment to assess adolescents’ attachment relationships with their parents. Developed by Armsden and Greenberg (1987), the questionnaire comprises two subscales: paternal and maternal attachment. Each subscale contains 10 items rated on a 5-point scale (1 = “Not at all true,” 5 = “Very true”). Higher scores indicate greater attachment security. The questionnaire demonstrates good reliability and validity among Chinese adolescents (Yang et al., 2023). In this study, the *Cronbach’s α* coefficient for the maternal attachment subscale was 0.804, and for the paternal attachment subscale, it was 0.878, indicating good reliability and validity.

#### 2.2.2 Shyness

This study employed the Cheek and Buss Shyness Scale to assess adolescents’ levels of shyness. Developed by Cheek and Buss, the scale comprises 13 items (Hopko et al., 2005). A five-point Likert scale (1 = “Strongly disagree,” 5 = “Strongly agree”) was employed, with higher scores indicating greater levels of shyness. This scale has been extensively applied among Chinese adolescents and demonstrates sound reliability and validity (Bai et al., 2025; Wu et al., 2021). In this study, the *Cronbach’s α* coefficient for the scale was 0.866, indicating good reliability and validity.

### 2.3 Data analysis

Data analysis for this study was conducted using SPSS statistical software version 26.0. First, to examine the potential impact of common method bias, exploratory factor analysis was conducted on all questionnaire items using Harman’s single-factor test. The results revealed eight factors with eigenvalues greater than 1. The first common factor explained 21.19% of the total variance, falling below the 40% criterion, indicating that common method bias was not significant. Subsequently, descriptive statistical analysis, independent samples t-tests, and Pearson correlation analyses were conducted on key variables including maternal attachment, paternal attachment, and adolescent shyness to examine the distribution characteristics of these variables and their interrelationships.

During the primary analysis phase, the SPSS Response Surface Analysis (RAS) 4.0.4 macro was employed to conduct polynomial regression and response surface analysis (PRRSA) (Zongmanqiu, 2025), thereby examining the predictive role of maternal and paternal attachment consistency on shyness levels among adolescents with depressive disorders. To ensure comparability of variables and reduce multicollinearity among polynomial terms, all continuous variables included in the polynomial regression model (maternal attachment, paternal attachment, and shyness) were standardized using z-score transformation (M = 0, SD = 1). Meanwhile, following Fleenor’s recommendation (Fleenor et al., 1996), a threshold of 0.5 standard deviations was adopted to define horizontal differences: 112 mothers (44.44%) exhibited equal levels of maternal and paternal attachment, 64 mothers (25.40%) demonstrated higher maternal attachment than paternal attachment, and 76 mothers (30.16%) showed higher paternal attachment than maternal attachment. Age was examined in preliminary analyses and was not significantly correlated with the main study variables; therefore, it was not included as a covariate in the final polynomial regression model to maintain model parsimony. Gender was incorporated into the model as a control variable to account for potential gender-related effects.

Based on the analytical framework proposed by Edwards and Parry (1993), the following quadratic polynomial regression equation is constructed:

$Z=b_{0}+b_{1}X+b_{2}Y+b_{3}X^{2}+b_{4}\mathrm{XY}+b_{5}Y^{2}+e$ Where Z represents the standardized level of shyness in adolescents with depressive disorders; X denotes the standardized maternal attachment score; and Y denotes the standardized paternal attachment score; b_0_-b_5_ are regression coefficients; and e is the residual term. The response surface parameters (a₁–a₄) estimated through the model can be used to further explore the characteristics of parental attachment’s influence on shyness under conditions of consistency and inconsistency. The consistency line (X = Y) reflects the linear and curvilinear effects when parental attachment levels are consistent, while the inconsistency line (X = –Y) reflects the direction and intensity of the influence of parental attachment differences on shyness. Finally, a three-dimensional response surface plot was generated using Origin 2024 to visually represent the trend of shyness levels under the interaction of parental attachment. All statistical tests employed two-tailed analyses with a significance level set at *α* = 0.05.

## 3 Results

### 3.1 Descriptive statistics and correlation analysis

Table 1 presents the descriptive statistics and group differences in maternal attachment, paternal attachment, and shyness among adolescents with depressive disorders. The participants had a mean age of 15.44 years (SD = 1.69), including 55 males (21.8%) and 197 females (78.2%). Independent-sample t-tests revealed a significant gender difference in maternal attachment (*t* = 2.213, *p* < 0.05), with males reporting higher maternal attachment scores than females. However, no significant gender differences were found in paternal attachment (*t* = 1.291, *p* > 0.05) or shyness (*t* = −0.799, *p* > 0.05). Correlation analyses further indicated that age was not significantly related to maternal attachment (*r* = 0.034), paternal attachment (*r* = −0.026), or shyness (*r* = −0.049), indicating that these key variables did not vary significantly with age. Therefore, age was not included as a control variable in subsequent analyses. Given the significant gender difference in maternal attachment, gender was included as a control variable in the subsequent polynomial regression and response surface analyses to account for its potential association.

**Table 1:** The differences among sample characteristics, maternal attachment, paternal attachment, and shyness (*N* = 252).

Variable	*M* (SD)/*N* (%)	Maternal attachment			Paternal attachment			Shyness		
		*M* (SD)	*t*	*r*	*M* (SD)	*t*	*r*	*M* (SD)	*t*	*r*
Gender			2.213^*^			1.291			−0.799	
Male	55 (21.8)	32.53 (5.86)			28.56 (8.60)			42.93 (9.23)		
Female	197 (78.2)	30.31 (6.79)			26.94 (8.13)			44.00 (8.67)		
Age	15.44 (1.69)			0.034			−0.026			−0.049

^*^p < 0.05, ^**^p < 0.01, ^***^p < 0.001.

M, mean; SD, standard deviation.

Table 2 presents the means, standard deviations, and Pearson correlation coefficients of the main study variables. The results showed a significant positive correlation between maternal attachment and paternal attachment (*r* = 0.288, *p* < 0.001), indicating that adolescents with higher levels of maternal attachment also had higher levels of paternal attachment. Both maternal attachment (*r* = −0.191, *p* < 0.01) and paternal attachment (*r* = −0.207, *p* < 0.001) were significantly negatively correlated with shyness, indicating that higher levels of parental attachment were associated with lower levels of shyness among adolescents with depressive disorders.

**Table 2:** Correlations among the measured variables (*N* = 252).

Variables	1	2	3
Maternal attachment	1		
Paternal attachment	0.288^***^	1	
Shyness	−0.191^**^	−0.207^***^	1
*M*	30.79	27.29	43.77
SD	6.65	8.24	8.79

^*^p < 0.05, ^**^p < 0.01, ^***^p < 0.001.

M, mean; SD, standard deviation.

### 3.2 Polynomial regression analysis

Using the RSA 4.0.4 macro, a polynomial regression model was constructed with maternal attachment (X) and paternal attachment (Y) as predictors and shyness (Z) as the outcome variable to examine the association between parental attachment congruence and shyness in adolescents with depressive disorders. Gender was included as a control variable in the analysis. As shown in Table 3, the overall model was significant (*ΔR*^2^ = 0.032, *p* < 0.05). Maternal attachment (*B* = −0.180, *t* = −2.741, *p* < 0.01) and paternal attachment (*B* = −0.145, *t* = −2.249, *p* < 0.05) were both significantly negatively associated with shyness, indicating that higher levels of attachment with either parent were associated with lower levels of shyness. The interaction term (MA × PA) was not statistically significant (*B* = −0.091, *t* = −1.734, *p* > 0.05), while the quadratic effect of paternal attachment (PA^2^) was significant (*B* = 0.118, *t* = 2.574, *p* < 0.05). The quadratic effect of maternal attachment (MA^2^) was not significant (*B* = −0.054, *t* = −1.157, *p* > 0.05). These results indicate that both maternal and paternal attachment were significantly associated with adolescents’ shyness levels, and that the association between paternal attachment and shyness may be nonlinear.

**Table 3:** Polynomial regression analysis (*N* = 252).

Predictor	*B*	*SE*	*t*	Δ*R*^2^
Constant	−0.038	0.080	−0.471	0.032^*^
Maternal attachment(MA)	−0.180	0.066	−2.741^**^	
Paternal attachment(PA)	−0.145	0.064	−2.249^*^	
MA^2^	−0.054	0.047	−1.157	
MA × PA	−0.091	0.052	−1.734	
PA^2^	0.118	0.046	2.574^*^	
Gender	0.031	0.063	0.508	

Include gender as a covariate in the analysis, ^*^p < 0.05, ^**^p < 0.01, ^***^p < 0.001.

### 3.3 Response surface analysis

**Figure 1:**
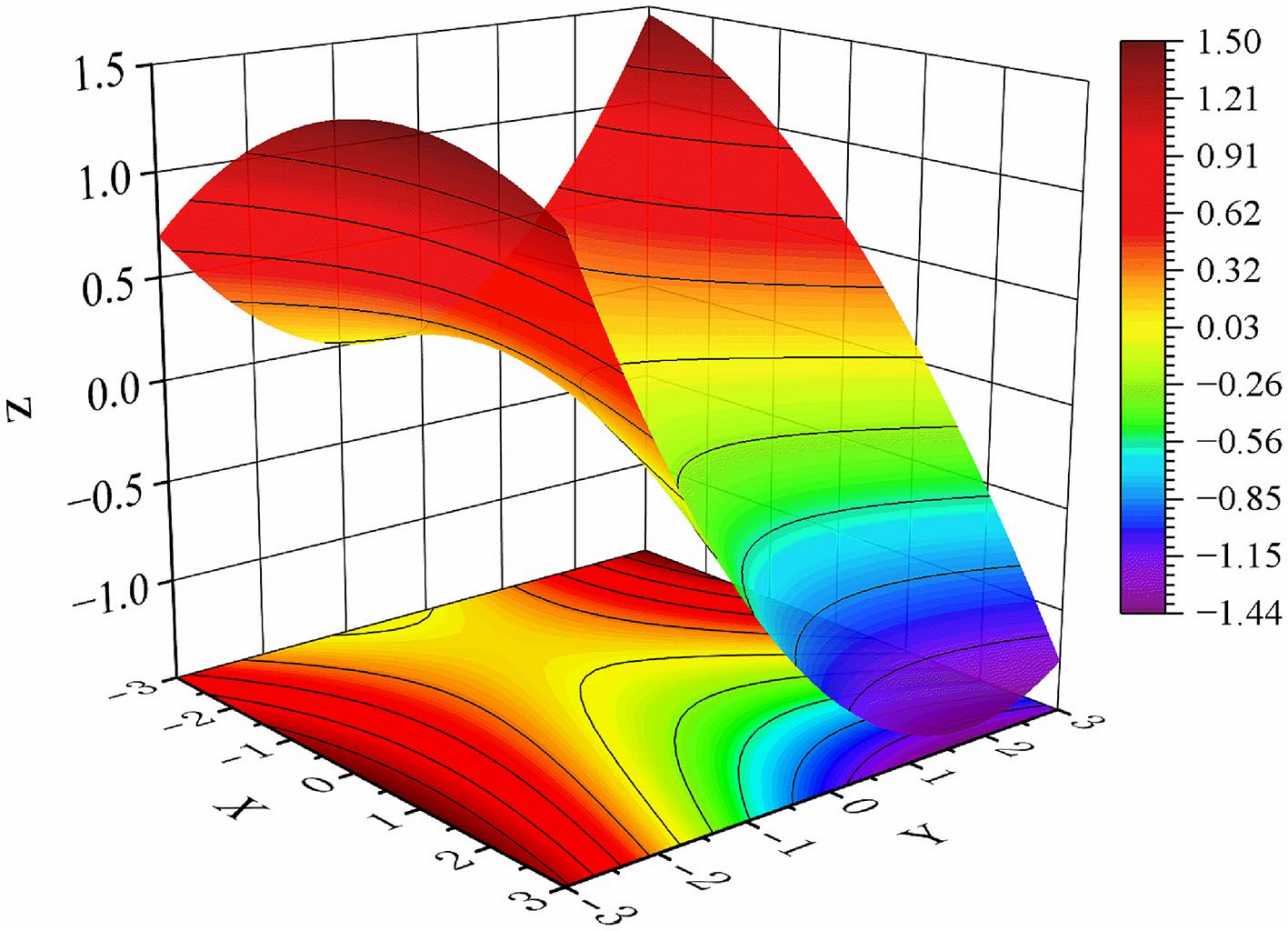
Response surface plot illustrating the relationship between maternal attachment, paternal attachment, and shyness in adolescents with depressive disorders. The *X*-axis represents standardized maternal attachment, the *Y*-axis represents standardized paternal attachment, and the *Z*-axis represents standardized shyness scores (*z*-scores; M = 0, SD = 1). The color gradient corresponds to the magnitude of predicted shyness values, with the color bar indicating the exact range of standardized scores.

In this study, maternal attachment (X) and paternal attachment (Y) were used as predictor variables, and shyness (Z) was set as the outcome variable to establish a response surface model examining the association between parental attachment congruence and shyness in adolescents with depressive disorders. As shown in Table 4 and Figure 1, along the line of congruence (X = Y), the linear effect a_1_ was significantly negative (a_1_ = −0.325, *t* = −4.164, *p* < 0.001), indicating that adolescents exhibited significantly lower levels of shyness when both parents’ attachment security was high. Along the line of incongruence (X = -Y), the linear effect a_3_ was not significant (a_3_ = −0.036, *t* = −0.342, *p* > 0.05), suggesting that the direction of discrepancy between maternal and paternal attachment was not significantly associated with shyness.

**Table 4:** Response surface analysis (*N* = 252).

Shyness	*B*	*SE*	*t*
a_1_	−0.325	0.078	−4.164^***^
a_2_	−0.027	0.061	−0.437
a_3_	−0.036	0.104	−0.342
a_4_	0.155	0.089	1.730

^*^p < 0.05, ^**^p < 0.01, ^***^p < 0.001.

## 4 Discussion

In recent years, the prevalence of depressive disorders among Chinese adolescents has continued to rise, emerging as a major public health concern that is associated with adverse impacts on their mental health and social adaptation. Examining the factors associated with shyness in this population is therefore of considerable importance. Shyness is closely linked to depressive symptoms and impaired social functioning in adolescents. Previous studies have demonstrated a significant association between parent–child attachment and adolescent shyness. However, most research has treated parent–child attachment as a single composite construct, rarely distinguishing the independent roles of maternal and paternal attachment. In addition, the potential role of consistency between maternal and paternal attachment in relation to adolescent shyness has been largely overlooked. Based on these gaps, the present study systematically examined the associations between maternal and paternal attachment consistency and shyness among adolescents with depressive disorders, using polynomial regression and response surface analysis. The results show that both maternal and paternal attachment are significantly negatively associated with shyness. Adolescents reported the lowest levels of shyness when both parents exhibited high and consistent attachment security. Overall, the findings are consistent with our hypotheses.

This study found that both maternal attachment and paternal attachment were significantly and negatively associated with shyness in adolescents with depressive disorders. In other words, higher levels of attachment security with both parents were associated with lower levels of shyness. This finding is consistent with previous research (Mattanah et al., 2011; Barstead et al., 2018), suggesting that secure parent–child attachment is associated with adolescents’ socio-emotional development. According to attachment theory (Ainsworth, 1997), secure attachment is associated with a stable sense of emotional support and safety, which may be related to more effective regulation of anxiety in situations involving social evaluation or unfamiliar environments, and may be associated with lower tendencies toward shyness. The negative association between maternal attachment and shyness may reflect the central role of mothers in emotional communication and acceptance. Previous studies have shown that mothers are often reported as the primary source of emotional support in parent–child relationships, and that maternal warmth and sensitivity are associated with the development of secure attachment (Rothenberg et al., 2020). Adolescents with secure maternal attachment are therefore more likely to report trust and acceptance in social interactions, which is associated with lower levels of shyness. In contrast, the negative association between paternal attachment and shyness may be related to the father’s role in promoting autonomy and social exploration. A secure father–child attachment is often associated with behavioral models and is considered to be related to adolescents’ confidence and curiosity in social contexts (Grossmann et al., 2002; Dumont and Paquette, 2013). Taken together, maternal emotional support and paternal encouragement of autonomy may be associated with adolescents’ social self-efficacy and emotional stability, and may be related to lower levels of shyness through distinct but complementary pathways.

The study found a significant nonlinear association between paternal attachment and adolescent shyness, indicating a U-shaped pattern. Specifically, both low and excessively high levels of paternal attachment were associated with higher levels of shyness, whereas moderate levels were associated with lower levels of shyness among adolescents with depressive disorders. This finding extends attachment theory by suggesting that the association between paternal attachment and socio-emotional functioning may not be strictly linear, but may instead follow a curvilinear pattern. From a family systems perspective, fathers contribute to adolescent development through both emotional security and autonomy support (Rothbaum et al., 2002). Consistent with this view, insufficient paternal attachment may be associated with lower levels of adolescents’ perceived emotional security and interpersonal confidence, and may be related to increased vulnerability to social inhibition and shyness (Li et al., 2022). The interpretation of the association observed at high levels of paternal attachment should be made cautiously. Because constructs such as overprotection or psychological enmeshment were not directly assessed in the present study, no specific mechanism can be empirically confirmed. Nevertheless, one possible explanation is that very high levels of paternal attachment may, in some family contexts, be associated with reduced opportunities for autonomy development and independent social engagement. From the perspective of self-determination theory, limited autonomy support may be associated with less optimal self-regulation and higher sensitivity to social evaluation (Flannery, 2017), which could in turn be associated with higher levels of shyness in interpersonal situations. Taken together, the observed U-shaped pattern suggests that moderate levels of paternal attachment may be most adaptive for adolescent socio-emotional functioning. These findings highlight the potential importance of balancing emotional support and autonomy granting in father–child relationships among adolescents with depressive disorders.

This study further found that when maternal and paternal attachment security were both high, adolescents with depressive disorders showed lower levels of shyness. These findings primarily highlight the association between overall parental attachment security and adolescents’ socio-emotional functioning, rather than indicating a differential association of parental attachment discrepancy. This finding provides partial support for the Attachment Matching Hypothesis (Cowan and Cowan, 2002). When parents maintain consistency in emotional support and parenting style, they may be associated with a more stable and predictable attachment environment. This stability may be associated with adolescents’ perceived emotional security toward others and may be related to lower levels of anxiety and shyness in social situations. From a family systems perspective (Rothbaum et al., 2002), parents function as joint attachment figures. Coordination between their emotional responses and parenting behaviors may be associated with reduced cognitive conflict and emotional ambiguity in parent–child interactions. Such consistent emotional experiences are considered to be associated with the development of a more stable internal working model. Importantly, this association may be particularly relevant in the context of depressive disorders. According to cognitive theories of depression, individuals with depression are characterized by a negative cognitive triad involving negative views of the self, the world, and the future (Golonka et al., 2024). A stable and secure internal working model, associated with consistent parental attachment, may be related to less pronounced maladaptive cognitive patterns in several ways. Specifically, secure attachment relationships may be associated with a more positive sense of self-worth, which is related to less negative self-evaluations (Reis et al., 2025). At the same time, consistent and supportive caregiving experiences may be associated with more trusting expectations of others, which may be related to lower interpersonal vigilance and social anxiety (Dykas and Cassidy, 2011). Furthermore, a predictable and emotionally secure family environment may be associated with adolescents’ perceived sense of future stability and control, which may be related to lower feelings of hopelessness (Lin et al., 2024). Through these pathways, a secure internal working model may be associated with buffering of depressive cognitive biases and, in turn, may be related to lower levels of shyness and social withdrawal in adolescents with depressive disorders. This stable internal working model may further be associated with adolescents’ social trust and self-worth, which may be related to greater confidence in social situations.

In addition, the present study did not find a significant association between the discrepancy between maternal and paternal attachment and adolescent shyness. This finding suggests that, within this clinical sample of adolescents with depressive disorders, inconsistency between maternal and paternal attachment security may not be a prominent correlate of shyness. One possible explanation is that adolescents with depressive disorders are characterized by heightened negative emotional processing and social withdrawal tendencies (Fernández-Theoduloz et al., 2019; Clifford et al., 2022). In this context, their social functioning may be more strongly associated with general emotional vulnerability and impaired emotion regulation, which may be related to a reduced association between discrepancies in maternal and paternal attachment and shyness. This result also suggests that, for this population, the absolute level of attachment security—particularly the presence of at least one highly secure attachment relationship—may be more relevant for social adjustment than the degree of consistency between parents. In other words, sufficient emotional support from one caregiver may be associated with weaker associations between inter-parental inconsistency and shyness. From a theoretical perspective, this finding differs from traditional assumptions emphasizing the importance of parental consistency, and instead highlights the potential relevance of individual affective regulation processes in adolescents with depressive symptoms. It is also possible that depressive symptomatology is associated with the relationship between parental attachment discrepancies and shyness, such that higher symptom severity may be associated with reduced sensitivity to differences between maternal and paternal attachment (Chan et al., 2023).

This study has several strengths. First, it is among the few empirical studies to systematically examine the association between maternal–paternal attachment consistency and shyness in adolescents with depressive disorders. This extends the scope of research on parent–child attachment and socio-emotional adjustment. Most previous studies have focused on general or community samples, whereas the present study targeted a clinical population, thereby providing findings with potential clinical relevance for understanding the role of attachment processes in psychopathological development. Second, this study employed polynomial regression and response surface analysis, which overcome the limitations of traditional difference and interaction approaches. These methods allow for a clearer differentiation between consistency patterns and directional inconsistency patterns, offering a more precise understanding of how parental attachment matching patterns are associated with shyness. Third, the inclusion of both maternal and paternal attachment dimensions enriches the family systems perspective within attachment theory, highlighting the complementary roles of both parents in adolescents’ socio-emotional development. Finally, the findings may have implications for clinical practice. The results suggest that secure parental attachment is associated with lower levels of shyness in adolescents, providing a theoretical basis for family-based approaches in the context of depressive disorders. Future interventions may consider focusing on strengthening parent–child attachment, improving family communication, and enhancing emotional support to support adolescents’ social functioning and emotional adjustment.

Despite its innovation and valuable insights, this study has several limitations. First, the research adopted a cross-sectional design, which precludes causal inferences regarding the association between parental attachment and shyness. Future studies may consider employing longitudinal or experimental designs to further examine the temporal associations between attachment consistency and changes in shyness over time. Second, data were collected using self-report questionnaires, which may be subject to response bias. Future research may consider incorporating multiple data sources, such as parent reports, teacher evaluations, or behavioral observations, to improve measurement validity and objectivity. Third, the sample was drawn from a single regional hospital in Ningxia, where cultural and socioeconomic backgrounds are relatively homogeneous. This may limit the generalizability of the findings. Future studies may consider replicating these findings in more diverse cultural and regional contexts. In addition, this study focused only on the attachment security dimension and did not distinguish between different attachment styles, such as anxiety and avoidance. This limits a more nuanced understanding of the potential differences underlying attachment subtypes. Finally, depressive symptom severity was not assessed in this study. Moreover, other potential confounding variables, including comorbid anxiety symptoms, illness duration, family functioning, and treatment status, were not systematically measured or statistically controlled. Therefore, their potential influence on the observed associations cannot be ruled out. Future research may consider incorporating these variables, including standardized assessments of depressive symptom severity, to provide a more comprehensive understanding of the associations among attachment, shyness, and depressive symptoms.

## 5 Conclusion

This study examined the associations between maternal and paternal attachment and shyness in adolescents with depressive disorders using polynomial regression and response surface analysis. The findings showed that both maternal and paternal attachment were negatively associated with shyness, suggesting that secure parent–child attachment may be associated with better socio-emotional adjustment in this clinical population. In addition, adolescents reported lower levels of shyness when both maternal and paternal attachment security were high, highlighting the potential relevance of a supportive family attachment environment for adolescents’ social functioning. Although discrepancies between maternal and paternal attachment were not significantly associated with shyness, the findings suggest that overall parental attachment security may be more relevant than directional differences between parents in understanding shyness among adolescents with depressive disorders. Overall, these findings underscore the potential association between supportive emotional relationships and coordinated parenting and adolescents’ socio-emotional adjustment. The findings may also provide a theoretical basis for family-based approaches aimed at improving parent–child attachment relationships and supporting psychosocial outcomes in adolescents with depressive disorders.

## Data Availability

The raw data supporting the conclusions of this article will be made available by the authors, without undue reservation.
